# 

**Published:** 1872-10

**Authors:** 


THREE DOLLARS A YEAR. SPECIMEN COPIES, 30 CENTS.

VOL. XXIX. October, 1872. NUMBER 10.

THE

### The Chicago Medical Journal.

J. ADAMS ALLEN, M.D., LL.D., WALTER HAY, M.D.,

EDITORS.

CHICAGO:

W. B. KEEN, COOKE & CO., PUBLISHERS,

NO. 408 WABASH AVENUE.

*The postage on this Journal is 24 cents a year—payable quarterly in advance.*

